# Negative effects of the COVID-19 pandemic on nurses can be buffered by a sense of humor and appreciation

**DOI:** 10.1186/s12912-021-00770-5

**Published:** 2021-12-20

**Authors:** Marek Bartzik, Fabienne Aust, Corinna Peifer

**Affiliations:** Department of Psychology, Research Group Work and Health, University of Lübeck, Lübeck, Germany

**Keywords:** COVID-19, Sense of humor, Appreciation, Flow experience, Satisfaction, Health care nurses, Geriatric nurses

## Abstract

**Background:**

The first analyses of the various consequences of the COVID-19 pandemic show that the risk to nurses’ psychological well-being is particularly high. As the pandemic and the demands imposed on nurses are not yet fully understood, there is a need to seek buffering factors to protect nurses’ psychological health. In line with the earliest evidence, we hypothesize pandemic-related increases in perceived stress and decreases in the frequency of flow experiences, likewise in satisfaction with work, life, work performance, and well-being. As protective factors while dealing with pandemic-related stress, we suggest an individual’s sense of humor and perceived appreciation.

**Methods:**

In June/July 2020 – during the first lockdown in Germany – participants completed an online-survey in which they were asked to rate their situation before the pandemic (retrospectively) and during the pandemic. Our sample consisted of 174 registered nurses (161 females, 13 males, *M*_age_ = 40.52), of whom 85 worked as public health nurses and 89 as geriatric nurses.

**Results:**

During the pandemic, nurses felt more stressed, had fewer flow experiences, and were less satisfied with their work, life, work-performance, and well-being than before the pandemic. In addition, nurses felt more appreciation from society but less from their patients. Sense of humor and the perceived appreciation of society and patients were confirmed as buffers of negative pandemic-related effects.

**Conclusion:**

Our study contributes to the so far scarce knowledge on nurses’ pandemic-related stress and well-being in combination with their resources. Moreover, we were able to identify sense of humor and appreciation as protective factors.

**Supplementary Information:**

The online version contains supplementary material available at 10.1186/s12912-021-00770-5.

## Introduction

At the end of 2019, the coronavirus (SARS-CoV-2) broke out in Wuhan (China) and quickly spread around the world. The outbreak of the coronavirus and its worldwide spread was considered by the World Health Organization to have reached the level of a pandemic in March 2020 [1]. Since the beginning of the outbreak until 12/06/2020 (2:48 pm CET), 65,870,030 people have been confirmed to have been infected worldwide and 1,523,583 people have died as a result of the pandemic [2]. Health care systems have been particularly severely affected by the COVID-19 pandemic. Therefore the nursing profession has come increasingly into focus. In Germany, these occupations are labeled as systemically relevant, meaning that even in such a worldwide crisis their work is indispensable. First analyses of the consequences of the COVID-19 pandemic confirm that the risk to nurses’ psychological well-being is currently particularly high: Chinese nurses with close contact to infected patients were twice more likely to suffer from anxiety and depression than were non-clinical staff [3]. A second study on nurses from 34 Chinese hospitals reported an alarming prevalence of depression (50.4%), anxiety (44.6%), insomnia (34%) and distress (71.5%), with the highest prevalence in frontline health care [4]. Therefore, our study deals with the subjectively perceived psychological states of nurses before the COVID-19 pandemic compared to during the COVID-19 pandemic, focusing on perceived stress, frequency of flow experiences, work and life satisfaction, and satisfaction with work performance and wellbeing. We moreover look at the resources that help to deal with the special demands during these difficult times; more specifically, we are interested in factors protective against of these psychological states such as sense of humor and appreciation.

### Stress

An important variable that should be considered in the context of care during the COVID-19 pandemic is stress. According to the transactional model of stress and coping by Lazarus and Folkman [5], there is a primary appraisal of the stressor, in which the stressor is classified as positive, negative, or irrelevant for a person. In case of a negative assessment of the stressor, a secondary appraisal is carried out, which compares the available resources with the demands. If the demands exceed the available resources, the person perceives stress. There are correlations between somatic symptoms in nurses and their perceived stress [6], which makes it even more important to investigate the relationships between different stressors in the care context and the subjective perceptions of stress. Another negative outcome of work-related stress is burnout, which also occurs among nurses [7, 8]. In the care context, numerous stressors, such as direct contact with patients, too little time to perform duties adequately or an uncongenial work environment have been identified as causing stress [9]. During the COVID-19 pandemic, additional stressors have been reported, such as the increased workload due to increased hygiene regulations and requirements to perform COVID-19 tests – or the psychological stressors related to the fear that family members will be infected [10, 11]. In line with this, over 80% of participants in a questionnaire study on nurses reported that they experienced stress during the COVID-19 pandemic [12]. This finding was also confirmed by a review including 59 studies [13]. In this study, we would like to add to this research, asking participants about their subjective stress experiences before and during the COVID-19 pandemic. Based on earlier research, we hypothesize that *the nurses will report an increase in perceived stress during the COVID-19 pandemic compared to before the COVID-19 pandemic (Hypothesis 1).*

### Flow experience

Another variable that is interesting to investigate is the frequency of flow experience. Flow is described as a pleasant and rewarding state of full absorption when performing activities which provide clear feedback, clear goals, and a balance between demands and abilities [14]. While there is a lot of research on flow in the work context [15, 16], it has rarely  been considered in the context of nurses. However, flow is associated with many positive work-related outcomes, such as increases in positive affect [17–19] and decreases in negative affect [17]. Flow is positively associated with job performance, job satisfaction, well-being, work engagement, organizational commitment, and also reducing the subjective perception of stress [15, 16, 20–22]. Besides these positive work-related outcomes, research shows some association of flow experience with stress [23, 24]. In particular, it was found that stress-related physiological indicators are related to flow in an inverted u-shaped way [23, 25, 26]. This means that, compared to a condition of boredom or relaxation, flow is associated with moderate increases in stress-related physiological parameters. Higher levels of physiological activation are again associated with lower levels of flow and are rather an indicator for stress. Due to the COVID-19 pandemic, nurses stress levels rose [12, 13, 27, 28], so that the level of moderate physiological activation was most likely often exceeded. Accordingly, we suspect that the nurses experienced less flow in their daily work during the COVID-19 pandemic than before the COVID-19 pandemic. We hypothesize that *nurses experienced less frequent flow during the COVID-19 pandemic than before the COVID-19 pandemic (Hypothesis 2).*

### Satisfaction with work, life, work performance, and well-being

Finally, in this study, the nurses’ satisfaction with their work, lives, work performance and well-being before and during the COVID-19 pandemic was investigated. It may be that satisfaction with work changes for the worse due to stressful working conditions and new procedures for hygiene and COVID-19 testing. There is already evidence of impaired work satisfaction due to the COVID-19 pandemic [29]. We also postulate that satisfaction with life deteriorates because work satisfaction and life satisfaction are closely linked [30]. The first results during the COVID-19 pandemic show a decline in life satisfaction [31, 32]. Similar effects are predicted for satisfaction with work performance. Caring for patients with COVID-19 may also have an influence on nurses’ satisfaction with their level of well-being [33]. Due to the risk posed by coming into contact with COVID-19 patients, nurses could be less satisfied with their well-being than they were before the COVID-19 pandemic. We hypothesize that the *nurses will report lower satisfaction with work (Hypothesis 3a), satisfaction with life (Hypothesis 3b), satisfaction with work performance (Hypothesis 3c), and satisfaction with well-being (Hypothesis 3d) during the COVID-19 pandemic than before the COVID-19 pandemic.*

### Protective factors

Due to their stress-protective effects found in earlier studies, we want to investigate sense of humor and appreciation as resources that could reduce the negative effects of the COVID-19 pandemic on nurses’ perceived stress, frequency of flow experience, and their satisfaction with work, life, work performance and well-being.

### Buffering effect of a sense of humor

The initial evidence shows that nurses successfully used humor as a coping strategy during the COVID-19 pandemic [34]. The construct of humor is a concept from Positive Psychology [35] and one of the 24 character strengths defined by Peterson and Seligman [36]. Sense of humor was found to be a protective factor against anxiety and depression [37] and was also found to be protective in adverse circumstances [38]. It can be defined as:

"(…) a habitual behavior pattern (tendency to laugh frequently, to tell jokes and amuse others, to laugh at other people’s jokes), an ability (ability to create humor, to amuse others, to “get the joke,” to remember jokes), a temperamental trait (habitual cheerfulness), an aesthetic response (enjoyment of particular types of humorous material), an attitude (positive attitude toward humor and humorous people), a world view (bemused outlook on life), or a coping strategy (tendency to maintain a humorous perspective in the face of adversity)" [39, p. 315].

Sense of humor can be divided into six humor habits [40]. These are: enjoyment of humor, laughter, verbal humor, finding humor in everyday life, laughing at yourself, and humor under stress [41, 42]. There is evidence that the use of humor can increase individuals’ well-being [43–45]. Humor can moreover serve as a coping strategy in the transactional model of stress and coping by Lazarus and Folkman [5]. Through cognitive appraisal and the resulting behavior, humor can be used as a coping strategy [46]. The use of humor creates positive emotions [43, 47, 48] that are incompatible with stress and thus lead to coping [49]. Fun and playfulness are described as factors conducive to flow in everyday work [50–52]. Hence, we also expect positive effects of sense of humor on flow, although this relationship has not so far been investigated. We therefore hypothesize that *sense of humor, as a coping strategy, can serve as a buffer, which reduces the negative effects of the COVID-19 pandemic on perceived stress (Hypothesis 4a), frequency of flow experience (Hypothesis 4b), satisfaction with work (Hypothesis 4c), satisfaction with life (Hypothesis 4d), satisfaction with work performance (Hypothesis 4e), and satisfaction with well-being (Hypothesis 4f).*

### Appreciation

One definition of appreciation is “acknowledging the value and meaning of something—an event, a person, a behavior, an object—and feeling a positive emotional connection to it.” [53, p. 81]. The COVID-19 pandemic has focused attention on the healthcare sector, especially on nurses. Clapping from apartment balconies for nurses was established in many cities as a sign of appreciation, and there were also monetary bonuses for nurses. We assume that these signs of appreciation led to nurses having a subjective feeling of increased appreciation from society as well as from direct interaction with patients. In line with this, a qualitative study regarding the effects of the COVID-19 pandemic found that nurses reported they would work with a state of appreciation in the future [34]. In a first step, we would like to add to this qualitative result and investigate quantitatively if health nurses’ subjective perceptions of appreciation for their work has increased due to the COVID-19 pandemic. Based on the preliminary findings, we hypothesize that *subjective perceived appreciation among nurses’ changes for the better during the COVID-19 pandemic (Hypothesis 5).*

### Buffering effect of appreciation

While assuming that the perception of appreciation has changed for the better during the COVID-19 pandemic, we also suggest that this can act as a resource, buffering the negative effects of the COVID-19 pandemic. Supporting this assumption, it was be shown that managers’ appreciation of their employees is positively associated with well-being and job satisfaction, and negatively associated with job-related depressive mood and sleep problems [54]. Feedback can be a form of appreciation. As shown in a meta-analysis, feedback has positive effects on performance, and this was especially the case with positive feedback [55]. One possible mechanism is the increase in self-efficacy [56]. Hence, our hypothesis is that *appreciation can serve as a buffer which reduces the negative effects of the COVID-19 pandemic on perceived stress (Hypothesis 6a), frequency of flow experience (Hypothesis 6b), satisfaction with work (Hypothesis 6c), satisfaction with life (Hypothesis 6d), satisfaction with work performance (Hypothesis 6e), and satisfaction with well-being (Hypothesis 6f).*

## Methods

### Participants

The sample was recruited through postings on social networks, especially in groups with public health nurses and geriatric nurses. We moreover contacted institutions with health care nurses and geriatric nurses directly via e-mail and asked them to disseminate information on the survey. The questionnaire was online from 06/01/2020 until 07/31/2020. In total 299 participants started to fill out the online questionnaire, but 125 did not complete it and were excluded from the analysis. The final sample consisted of 174 registered nurses (161 females, 13 males). Eighty-five worked as public health nurses and 89 as geriatric nurses. The participants had completed a three-year training program with a state examination (In our sample size, four public health nurses and 11 geriatric nurses were currently in training). Due to missing data and the exclusion of outliers on all variables involved in the analysis (+/− 2.5 *SD*) *n* varies between 152 and 174 for the different analyses. The mean age was 40.52 (*SD*_*age*_ = 10.75) and ranged between 18 and 62 years. Two participants skipped the question about their ages and 147 provided information about their professional experience. On average the participants had 18.65 years (*SD*_*experience*_ = 10.90) of experience in their profession, the range being between one year and 43 years.

### Procedure

During the COVID-19 pandemic, we created an online questionnaire that can be divided into five parts: (1) In the demography data section, we elicited demographic information on the participants (e.g., age, gender, work experience). (2) Next we asked about their sense of humor. (3) Then we introduced questions on subjective experience before the COVID-19 pandemic (t1) with the following instruction: “*Now please put yourself in the time at the beginning of February this year before the corona pandemic. The year had already started a few weeks ago, Christmas and New Year’s Eve were felt to be long gone. The weather was clearly too warm, too windy, too wet, and with too little sunshine for the taste of the meteorologists. There were the first evenings when it grew dark a little later. At work the daily business was in full progress...Please put yourself in the position you were in before the corona crisis, at the beginning of February 2020, and answer the following questions.*”. (4) After the block of questions on subjective perception before the COVID-19 pandemic came questions on subjective perception during the COVID-19 pandemic (t2). We introduced the section with the instruction: “*Please revert to your situation in your everyday work during the corona pandemic and answer the following questions.*”. (5) Finally, we asked three questions about concerns regarding the COVID-19 pandemic.

#### Measures

Self-constructed scales and items are provided in English and German in the Supplementary Material - Table S1.

### Stress

We used three different measurements for mental stress. First, we used one single item (“How stressed did you feel?”) to measure the participants’ stress. They rated their stress levels on a 5-point rating scale from 1 = *not at all* to 5 = *very strong.* Second, stress was measured with the subscale emotional irritation of the *Irritation Scale* by Mohr and colleagues [57]. Participants rated the five items on a 7-point rating scale from 1 = *do not agree at all* to 7 = *totally agree.* One example item is “Even at home I often think of my problems at work”. The reliabilities for the two measurement times were good (Cronbach’s α = .84 (t1) or .90 (t2)). Third, we used the subscale for emotional exhaustion of the German version (*MBI-D)* by Büssing and Perrar [58] of the *Maslach Burnout Inventory* [59]*.* Participants were asked to rate nine items (e.g., “Working with people all day is really a strain for me”) on a 7-point rating scale from 1 = *never occurred* to 7 = *occurred often.* The Cronbach’s α were very good with .92 at t1 or .93 at t2.

### Flow experience

Flow experience was measured with the recently developed *Flow Frequency Scale* by Bartzik and Peifer (in preparation) [60]. The scale consists of ten items and can be divided into three subscales. These are: absorption (e.g., “How often did you experience at work that you were completely focused on what you were doing?”), perceived demand-skill balance (e.g., “How often did you experience at work that you could use your skills to the optimal extent”), and enjoyment (e.g., “How often did you experience at work that you felt joy in what you were doing”). Participants rated how often they had those experiences on a 6-point rating scale from 1 = *never* to 6 = *(almost) always.* We found good to questionable reliabilities for absorption (Cronbach’s α = .62 (t1) or .71 (t2)), perceived demand-skill balance (Cronbach’s α = .86 (t1) or .89 (t2)), and enjoyment (Cronbach’s α = .91 (t1) or .94 (t2)). Cronbach’s α for the full scale was .93 at t1 and .94 at t2.

### Satisfaction

Satisfaction was measured with four self-constructed single items – satisfaction with work, life, professional performance, and well-being. The participants rated their satisfaction on a 7-point rating scale from 1 = *extremely dissatisfied* to 7 = *extremely satisfied.* An example item is “All in all, how satisfied were you with your work?”. The different points on the scale additionally provided smileys to support the decision.

### Sense of humor

To measure sense of humor we used the parallel form of the *Sense of Humor Scale* (*SHS-P*) by Ruch and Heintz [40]. The version used consists of 24 items rated on a 7-point rating scale from 1 = *strong disapproval* to 7 = *strong agreement*. Although the overall value of the scale (sense of humor), six subscales can be distinguished – enjoyment of humor (e.g., “I enjoy funny sketches”), laughter (e.g., “I like laughing, because it makes me feel good”) verbal humor (e.g., “I often make funny comments”), finding humor in everyday life (e.g., “I see funny occurrences in the daily routine”), laughing at yourself (e.g., “If something embarrassing happens to me, I can laugh about it”), humor under stress (e.g., “Even in difficult situations my humor does not leave me”). Cronbach’s α for the scale was .92. The Cronbach’s α for the subscales varied between .71 and .84.

### Appreciation

To assess appreciation, we developed two single items. The first item focused on appreciation experienced from the patients and the second item elicited appreciation experienced from society (“How much did you feel appreciated by the patients? / society?”). There was a 5-point rating scale from 1 = *not at all* to 5 = *very much.*

### COVID-19 pandemic items

We constructed three items to measure the subjective consequences of the COVID-19 pandemic on a 6-point rating scale from 1 = *do not agree at all* to 6 = *totally agree*. An example item is “I was very concerned about my own health because of Corona.”.

### Workload during the COVID-19 pandemic

We asked the participants about their actual workloads during the COVID-19 pandemic. The item was “Because of the COVID-19 pandemic I had to work …”. Participants could choose between 1 = *significantly less*, 2 = *less*, 3 = *just the same*, 4 = *more,* or 5 = *significantly more*.

#### Data analysis

The statistical analyses were performed with IBM SPSS statistics package V26. For all analyses we used pairwise deletion. To test Hypotheses 1, 2, 3, and 5 we performed two-tailed paired t-tests. Due to the large number of participants, a normal distribution can be assumed according to the central limit theorem [61]. We calculated Cohen’s *d*_*z*_ for paired samples manually. Regarding Hypotheses 4 and 6, we wanted to ascertain if sense of humor and appreciation can buffer against the negative effects of the COVID-19 pandemic on perceived stress, flow experience, work and life satisfaction, and satisfaction with work performance and well-being. Here we calculated difference scores of the outcome variables (*M*_during_ – *M*_before_) and performed linear regressions with sense of humor and the subscales and the difference scores on appreciation as independent variables. Therefore, we used linear regression models in SPSS.

## Results

### Descriptive data and Intercorrelations

To consider the exceptional circumstances during the COVID-19 pandemic we asked some general questions about the participants’ concerns. Participants were most concerned about the health of their family members and friends (*M* = 4.17, *SD* = 1.75). The fear of consequences to their own health was lower but still in the middle of the scale (*M* = 3.17, *SD* = 1.81). Concerns about their economic future were the lowest (*M* = 2.22, *SD* = 1.71). For an overview see Table 1.

**Table 1:** Means, minimum, maximum, and standard deviation of the COVID-19 pandemic items

Items	* n*	*Mean*	*Min.*	*Max.*	*SD*
Concerns about economic future	174	2.22	1.00	6.00	1.71
Concerns about the health of friends and family	174	4.17	1.00	6.00	1.75
Concerns about my own health	174	3.17	1.00	6.00	1.81

*Note.* COVID-19 pandemic items were measured on 6-point rating scale from 1 to 6

**Fig. 1:**
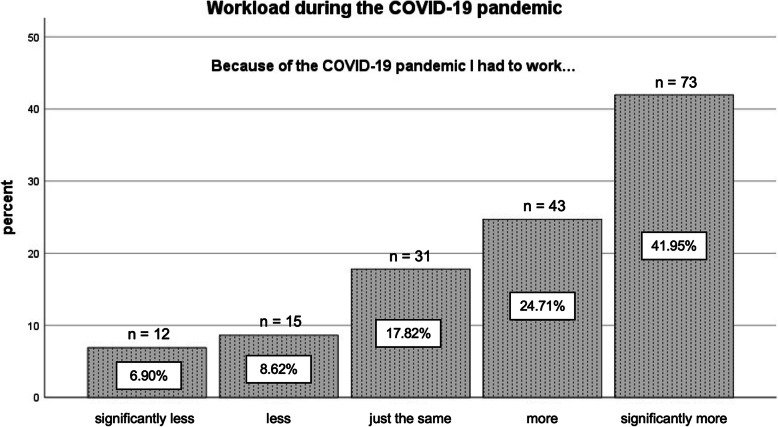
Workload during the COVID-19 pandemic. *Note.* Above the bars the frequencies of the nominations are indicated. *N* = 174

Figure 1 shows the change in workload due to the COVID-19 pandemic. It is evident that over 66% of the respondents had significantly more work or more work than before the COVID-19 pandemic. Only about 16% reported that they had significantly less or less to do. About 18% reported that their workload did not change due to the COVID-19 pandemic.

When examining the descriptive data on sense of humor it was noticeable that the mean values were relatively high. Participants reported high values (*M* = 6.03, *SD* = 0.93) in particular on the subscale *laughter*. For an overview see Table 2. An overview of the intercorrelations is given in the Supplementary Material (Table S2 to S4).

**Table 2:** Means, minimum, maximum, and standard deviation of the sense of humor

Scales	* n*	*Mean*	*Min.*	*Max.*	*SD*
Sense of humor	169	5.37	3.13	7.00	0.82
-Enjoyment of humor	169	4.99	1.75	7.00	1.24
-Laughter	168	6.03	3.25	7.00	0.93
-Verbal humor	170	5.14	2.00	7.00	1.15
-Finding humor in everyday life	169	5.27	2.50	7.00	1.03
-Laughing at yourself	170	5.63	3.00	7.00	1.05
-Humor under stress	173	5.17	1.75	7.00	1.35

*Note.* Sense of humor was measured on a 7-point rating scale from 1 to 7

#### Hypotheses 1, 2, 3, and 5

The COVID-19 pandemic affected nurses’ stress levels. They experienced more stress (*t* (173) = 3.14, *p* = .002, *d*_*z*_ = 0.24), had higher values on emotional irritation (*t* (171) = 4.63, *p* < .001, *d*_*z*_ = 0.35), and felt more emotionally exhausted (*t* (172) = 8.08, *p* < .001, *d*_*z*_ = 0.61) during the COVID-19 pandemic than during the time before COVID-19. These results support Hypothesis 1. The nurses also felt less flow during the COVID-19 pandemic than before (*t* (173) = − 7.67, *p* < .001, *d*_*z*_ = − 0.58), thereby confirming Hypothesis 2. This pattern was found for all subscales: absorption (*t* (169) = − 6.66, *p* < .001, *d*_*z*_ = − 0.51), perceived demand-skill balance (*t* (173) = − 5.38, *p* < .001, *d*_*z*_ = − 0.41), and enjoyment (*t* (172) = − 8.44, *p* < .001, *d*_*z*_ = − 0.64). Similarly, satisfaction with work (*t* (170) = − 5.91, *p* < .001, *d*_*z*_ = − 0.45), life (*t* (169) = − 5.91, *p* < .001, *d*_*z*_ = − 0.45), work performance (*t* (163) = − 6.69, *p* < .001, *d*_*z*_ = − 0.52), and well-being (*t* (166) = − 6.03, *p* < .001, *d*_*z*_ = − 0.47) also decreased during the COVID-19 pandemic, thereby confirming Hypothesis 3. Regarding appreciation from patients and society, we identified a pattern that was not entirely in line with our Hypothesis 5. While the nurses reported feeling more appreciation from society (*t* (166) = 3.54, *p* = .001, *d*_*z*_ = 0.27) during the COVID-19 pandemic (confirming Hypothesis 5), they felt *less* appreciation from their patients (*t* (173) = − 2.72, *p* = .007, *d*_*z*_ = − 0.21) during that time. All results concerning means, standard deviations, significance tests, and effect sizes are summarized in Table 3.

**Table 3:** Means, standard deviations, significance test and effect sizes before and during the COVID-19 pandemic

			Before the pandemic		During the pandemic				
Scales	Scale Range	* n*	*Mean*	*SD*	*Mean*	*SD*	* t*	*p* *two-tailed*	Cohen’s *d*_*z*_
Stress (single item)	1–5	174	3.16	0.98	3.46	1.09	3.14	** .002**	0.24
-Emotional irritation	1–7	172	2.55	1.39	3.12	1.65	4.63	**< .001**	0.35
-Emotional exhaustion	1–7	173	3.12	1.36	3.97	1.59	8.08	**< .001**	0.61
Flow	1–6	174	4.18	1.00	3.58	1.11	−7.67	**< .001**	−0.58
-Absorption	1–6	170	4.54	0.90	4.05	1.09	−6.66	**< .001**	−0.51
-Demand-skill balance	1–6	174	3.90	1.20	3.41	1.26	−5.38	**< .001**	−0.41
-Enjoyment	1–6	173	4.15	1.08	3.42	1.24	−8.44	**< .001**	−0.64
Satisfaction									
-Work	1–7	171	4.79	1.12	4.14	1.51	−5.91	**< .001**	−0.45
-Life	1–7	170	5.09	1.27	4.32	1.50	−5.91	**< .001**	−0.45
-Work performance	1–7	164	5.58	1.06	4.88	1.37	−6.69	**< .001**	−0.52
-Well-being	1–7	167	4.74	1.29	3.97	1.48	−6.03	**< .001**	−0.47
Appreciation									
-Patients	1–5	174	3.60	1.08	3.39	1.23	−2.72	** .007**	−0.21
-Society	1–5	167	2.01	0.99	2.37	1.23	3.54	** .001**	0.27

*Note.* Significant results (two-tailed; *p* < .05) are shown in bold face

#### Hypotheses 4 and 6

### Buffering effects of sense of humor

In Hypothesis 4 we postulated that sense of humor has a buffering effect on the different outcome variables during the COVID-19 pandemic. Participants scoring higher on the sense of humor scale were assumed to be less influenced by the COVID-19 pandemic than subjects with lower sense of humor values. We calculated the difference scores of all outcomes (*M*_during_ – *M*_before_) and performed a linear regression for the full scale and each subscale of the sense of humor scale.

Sense of humor (*R*^*2*^ = .04, β = −.20, *F* (1, 160) = 6.56, *p* = .011) and the subscales enjoyment of humor (*R*^*2*^ = .03, β = −.18, *F* (1, 160) = 5.51, *p* = .020), finding humor in everyday life (*R*^*2*^ = .05, β = −.21, *F* (1, 161) = 7.58, *p* = .007), and humor under stress (*R*^*2*^ = .06, β = −.25, *F* (1, 164) = 11.06, *p* = .001) buffered the effects of the COVID-19 pandemic on emotional exhaustion as expressed in significant effects on the difference scores. Nurses scoring higher on the humor facets had less increase in emotional exhaustion due to the COVID-19 pandemic. The same pattern was found for the effect of enjoyment of humor on emotional irritation (*R*^*2*^ = .04, β = −.20, *F* (1, 161) = 6.60, *p* = .011). Also as predicted, sense of humor (*R*^*2*^ = .03, β = .16, *F* (1, 163) = 4.19, *p* = .042), enjoyment of humor (*R*^*2*^ = .05, β = .23, *F* (1, 163) = 8.90, *p* = .003), finding humor in everyday life (*R*^*2*^ = .03, β = .16, *F* (1, 164) = 4.17, *p* = .043), and humor under stress (*R*^*2*^ = .07, β = .26, *F* (1, 167) = 12.35, *p* = .001) had significant effects on the difference scores of flow. Participants scoring higher on these subscales showed a smaller decrease of flow experience due to the COVID-19 pandemic than did subjects scoring lower on these sense of humor subscales. The subscale enjoyment of humor had a buffering effect on satisfaction with work (*R*^*2*^ = .05, β = .22, *F* (1, 161) = 8.03, *p* = .005) and humor under stress had a significant effect on satisfaction with work (*R*^*2*^ = .06, β = .25, *F* (1, 165) = 10.57, *p* = .001) and on satisfaction with work performance (*R*^*2*^ = .04, β = .20, *F* (1, 161) = 6.37, *p* = .013).

### Buffering effect of appreciation

In Hypothesis 6 we postulated that experienced change in appreciation due to the pandemic would have a buffering effect on stress, emotional irritation, emotional exhaustion, frequency of flow experience, and satisfaction. With the difference scores for appreciation as independent variables and the difference scores of the outcome variables we performed linear regressions. Appreciation from patients had a buffering effect on emotional exhaustion (*R*^*2*^ = .06, β = −.25, *F* (1, 162) = 10.44, *p* = .001), frequency of flow experience (*R*^*2*^ = .09, β = .31, *F* (1, 165) = 16.94, *p* < .001), satisfaction with work (*R*^*2*^ = .05, β = .22, *F* (1, 164) = 8.63, *p* = .004), and satisfaction with work performance (*R*^*2*^ = .06, β = .23, *F* (1, 159) = 9.25, *p* = .003). Appreciation from society only influenced frequency of flow experience (*R*^*2*^ = .04, β = .19, *F* (1, 159) = 5.83, *p* = .017). Stress (single item), satisfaction with life and well-being were not influenced by appreciation or sense of humor. Thus, we can only partially confirm Hypotheses 4 and 6. For an overview see Table 4.

**Table 4:** Buffering effects of sense of humor and appreciation using difference scores

	*Stress*		*Emotional* *Irritation*		*Emotional Exhaustion*		*Frequency of flow experience*		*Satisfaction –* *Work*		*Satisfaction –* *Life*		*Satisfaction –* *Work performance*		*Satisfaction –* *Well-being*	
	*R*^*2*^	β	*R*^*2*^	β	*R*^*2*^	β	*R*^*2*^	β	*R*^*2*^	β	*R*^*2*^	β	*R*^*2*^	β	*R*^*2*^	β
Appreciation																
-Patients	.01	−.11	.01	−.09	**.06**	−.25**	**.09**	.31**	**.05**	.22**	.00	−.05	**.06**	.23**	.00	.02
-Society	.00	−.04	.00	−.03	.00	−.03	**.04**	.19*	.00	.06	.02	−.14	.00	.01	.00	.06
Sense of humor	.01	−.11	.00	−.02	**.04**	−.20*	**.03**	.16*	.02	.13	.00	−.01	.00	.01	.00	−.03
-Enjoyment of humor	.01	−.08	**.04**	−.20*	**.03**	−.18*	**.05**	.23**	**.05**	.22**	.01	.07	.01	.12	.01	.10
-Laughter	.01	−.09	.01	.10	.00	−.06	.00	.03	.00	.04	.01	−.11	.00	−.01	.01	−.11
-Verbal humor	.01	−.08	.00	.01	.02	−.13	.02	.12	.02	.13	.01	.11	.00	.01	.00	.01
-Finding humor in everyday life	.00	−.03	.01	.12	**.05**	−.21**	**.03**	.16*	.01	.08	.00	−.03	.00	.05	.00	−.02
-Laughing at yourself	.02	−.14	.01	.08	.00	−.07	.00	.02	.00	.01	.02	−.12	.01	−.11	.01	−.12
-Humor under stress	.02	−.13	.00	−.06	**.06**	−.25**	**.07**	.26**	**.06**	.25**	.01	.11	**.04**	.20*	.01	.07

*Note.* Significant results are shown in bold face; ** *p* < .01; * *p* < .05. *n* varies due to the pairwise deletion of data between 152 and 169 (see Supplementary Material Table S5)

## Discussion

The aim of this study was to investigate effects of the COVID-19 pandemic on nurses’ subjectively perceived psychological states. We investigated *changes* in stress, frequency of flow experience, and satisfaction with work, life, work performance, and well-being during the COVID-19 pandemic compared to before the COVID-19 pandemic. We next examined the buffering effects of sense of humor and perceived appreciation on these psychological states. We could show that nurses felt more stressed, had flow experiences less frequently, and lower values of satisfaction with work, life, work performance and well-being during the COVID-19 pandemic compared to before the COVID-19 pandemic. They felt more appreciation from society but less from their patients. In line with our assumptions, we found both sense of humor and perceived appreciation to have buffering effects. More specifically, sense of humor buffered the negative effects of the COVID-19 pandemic for emotional exhaustion and frequency of flow experience. When looking more closely at its subscales, humor under stress buffers against the negative effects of the COVID-19 pandemic for emotional exhaustion, frequency of flow experience, satisfaction with work and satisfaction with work performance. Further, enjoyment of humor buffered the negative effects of the COVID-19 pandemic on emotional irritation, emotional exhaustion, frequency of flow experience, and satisfaction with work. Only the subscales laughter, verbal humor, and laughing at yourself of the sense of humor scale showed no buffering effects on any negative effects of the COVID-19 pandemic.

For perceived appreciation, we observed that perceived appreciation from patients had a buffering effect on emotional exhaustion, frequency of flow experience, satisfaction with work, and satisfaction with work performance. For perceived appreciation from society, only a buffering effect on frequency of flow experience was found.

In the following we discuss these results in light of further findings of our study and findings in the literature.

The heightened stress levels found in our study are in line with our further result that the COVID-19 pandemic had a massive influence on the workloads of the nurses in our sample. About 66% of the nurses stated that they had more or significantly more to do than before. Only 16% said that they had significantly less or less to do. These results show that the COVID-19 pandemic changed the working lives of nurses in Germany and underlines the importance of studies addressing the effects of the COVID-19 pandemic on employees, especially because workload is an important factor affecting nurses’ stress levels [9]. In line with this, increased workload during the COVID-19 pandemic could be one reason for the nurses’ increased stress. The increased stress levels in our study are also in line with results of a Chinese sample investigated in February and March 2020. In that study, a total of 97.9% of participants showed at least one posttraumatic stress symptom and about 40% were within the clinically relevant range (mild/positive). These rates are much higher than in the sample of university students who participated in the same study (94 and 34%). A total of 8.6% of the sample showed mild to extremely severe values of stress [62]. Further factors exacerbating the effects of the COVID-19 pandemic on the stress levels were, for example, confirmed COVID-19 cases within one’s living community, or among friends and relatives. Accordingly, this fear of infecting others was likely another reason for increased stress, and in particular for the increased emotional irritation found in our sample. This was supported by our descriptive results: With a mean of 4.17 (scale from 1 to 6), participants in our sample were concerned with the health of their family and friends. This result is even more alarming given that our study was conducted in June 2020, a time when the number of infections was relatively low in most parts of Germany. Similarly, the chances of getting a fatal disease were classified as a negative life event with a high negative valence [63]. Thus fear of contracting the disease oneself could be yet another factor with effects on stress. However, when looking at our descriptive results, fear of getting health issues oneself is rated lower (Mean = 3.17; scale from 1 to 6) than concern about the health of others. During the *Severe Acute Respiratory Syndrome (SARS)* breakout in 2003 nurses were emotionally affected [64]. This concurs with our findings here, that emotional irritation and emotional exhaustion increased due to the COVID-19 pandemic. At the same time, variables with a positive emotional tone decreased, i.e., frequency of flow experience, satisfaction with life, satisfaction with work, satisfaction with work performance, and satisfaction with well-being.

In terms of perceived appreciation, we identified an interesting pattern. While perceived appreciation from society increased, perceived appreciation from patients decreased. One reason may be the extraordinary situation in hospitals. Patients may have been frustrated due to the ban on visitors. Possibly they were in a bad mood and transferred these feelings to their nurses. By contrast, the nurses’ work came under the focus of society during the COVID-19 pandemic. People showed their respect by giving public applause, and politicians discussed giving a financial bonus. These factors may have influenced perceived appreciation from patients versus that from society. Gratitude for the support of society was already mentioned in a Chinese sample [34] and supports our result for the German sample in this study.

Frequency of flow experience decreased during the COVID-19 pandemic. As outlined in the introduction, we attribute this finding to the increased stress of nurses due to the COVID-19 pandemic [12, 13, 27, 28, 62]. This finding is in line with those of studies on the relationship between stress-related physiological indicators and flow experience: while moderate levels of stress are positively related to flow, high levels of stress were found to decrease flow [25, 26]. Another reason for the decreased flow experience could be that nurses had to change their working routines and had to learn new procedures, which meant that they could no longer use their existing expertise. In the context of flow it has been shown that experts experience more flow during an activity than do novices [65]. As the nurses had to learn new routines, their expert status possibly changed to novice status in some of their tasks.

Besides the negative main effect of the COVID-19 pandemic on the frequency of flow and the increasing effects on stress, we observed a buffering effect of sense of humor. This underlines the assumption that humor is a successful coping strategy [39], which should be fostered in difficult times. One reason for these buffering effects could be that humor in the workplace fosters cohesiveness among nurses [66]. In a study on the staff of a children’s blood and cancer center it was found that the feeling of belonging to a “work family” enhances resilience [67].

Our results showed different patterns for the subscales of the sense of humor scale. We therefore suggest investigating both: all subscales and the whole scale as recommended by the authors [40]. In addition to the buffering effects of a sense of humor, we also observed buffering effects of perceived appreciation by society and patients on frequency of flow experience.

### Strengths and limitations

Because we used an online questionnaire for our study, we were able to contact a large number of nurses and they were able to independently schedule their participation in this study. Another strength is that our study was conducted in the middle of the first lockdown in Germany, when the pandemic situation was ongoing among our target group. Also, by including many different psychological experiences (e.g., stress, flow experience, satisfaction, and appreciation) in our study and focusing on positive coping strategies, we were able to contribute to the development of ideas to better understand and help nurses in this challenging situation.

There are also some limitations that should be mentioned. First, this study was not longitudinal. The COVID-19 pandemic was unpredictable, so we used a cross-sectional approach. In order to still be able to assess the changes due to the COVID-19 pandemic, we used a retrospective approach and asked the nurses to relate their answers to the time before the COVID-19 pandemic started. We had good reason to hope that in June the time before the COVID-19 pandemic was still well remembered. However, we obviously cannot exclude the possibility of some recall bias. Because of the cross-sectional data, the assumption of causality cannot be statistically demonstrated. The other causal direction of the presented effects is also possible. While 299 participants started to fill out our online questionnaire, only 174 proceeded to the end of the survey. This high dropout rate may be another sign of the high strain these nurses were under.

### Implications and future research

More than half of Chinese nurses actively searched for psychological resources such as self-help coping methods and even psychotherapy during the COVID-19 pandemic [68]. This underlines the clear need for interventions that address nurses. Our results provide clear implications for such interventions.

The buffering effect of sense of humor (and its subscales) on stress, the frequency of flow and satisfaction underlines the high potential of using humor for stress management in the health care sector. Also, the use of humor was found to be appreciated by patients as a positive characteristic of nurses and is particularly important for nurse-patient interaction [69]. Hence, humor is additionally a potential approach to increase appreciation experienced from patients, and both are potential protective factors in everyday work. A promising approach is thus to cultivate a sense of humor in interventions for nurses through a targeted humor training [70]. An existing intervention is the “7 Humor Habits Program” by McGhee [42] that aims to build and strengthen humor in everyday life. Evidence for the effectiveness of this humor intervention in increasing humor has been reported in various studies [44, 71, 72].

It would moreover be possible to offer interventions that directly address the reduction of stress experience and the increase of flow experience. The literature suggests that stress can be transformed into flow experience [73] and further that flow can be used as a coping strategy [23, 74] and as a sustainer of coping [75]. Thus, specific training for nurses to increase flow in the work context would be beneficial for actively using flow as a coping strategy. Promoting flow in nurses is a promising approach to reducing negative stress.

In our study we were able to show the buffering effects of appreciation on stress, frequency of flow experience, and satisfaction. Therefore, it is important to increase appreciation for nurses from patients and from society. An approach to increasing patients’ appreciation and understanding is transparent information about the current situation so that they can better accept restrictions and not blame the nurses for it. Such information could be given in direct communication or information materials (e.g., flyers, information placards) provided by the hospital. Also, communication training may help nurses to communicate this information objectively but empathetically to patients. To further increase appreciation from society there should be information and awareness campaigns that underline the importance and the demands that are part of care workers’ profession so that people comprehend the important value of nurses.

## Conclusion

COVID-19 has rapidly changed the working conditions of nurses in Germany. This leads to an increase in stress level and a decrease in flow experiences, satisfaction, and appreciation from patients. Appreciation from society increased. Coping strategies are important to handle the COVID-19 pandemic among nurses. Sense of humor and appreciation are two resources that help nurses deal with the COVID-19 pandemic. Training in humor, training in communication, stress and flow experience offer a promising approach to dealing with the current challenges. More research on the working conditions of nurses and the effects of the COVID-19 pandemic on them is still needed.

## Supplementary Information

**Additional file 1:** **Table S1.** Self-constructed scales and items. **Table S2:** Pearson correlations between sense of humor and psychological states before and during the COVID-19 pandemic. **Table S3:** Intercorrelations before the COVID-19 pandemic. **Table S4:** Intercorrelations during the COVID-19 pandemic. **Table S5:** Buffering effects of sense of humor and appreciation using difference scores including sample sizes.

## Data Availability

The datasets used and/or analyzed during the current study are available from the corresponding author on reasonable request. E-Mail: marek.bartzik@uni-luebeck.de
