# Supplementary material for: Negative effects of the COVID-19 pandemic on nurses can be buffered by a sense of humor and appreciation
Source: BMC Nurs. 2021 Dec 20;20:257. doi: 10.1186/s12912-021-00770-5 (PMC8686101; doi:10.1186/s12912-021-00770-5)
Supplement: Supplementary file 1 — Additional file 1: Table S1. Self-constructed scales and items. Table S2: Pearson correlations between sense of humor and psychological states before and during the COVID-19 pandemic. Table S3: Intercorrelations before the COVID-19 pandemic. Table S4: Intercorrelations during the COVID-19 pandemic. Table S5: Buffering effects of sense of humor and appreciation using difference scores including sample sizes. [file 12912_2021_770_MOESM1_ESM.docx]

**Negative effects of the COVID-19 pandemic on nurses can be buffered by a sense of humor and appreciation**

**Supplementary File**

**Marek Bartzik^1*^, Fabienne Aust^1^, & Corinna Peifer^1^**

**^1^**University of Lübeck, Department of Psychology, Research Group Work and Health, Lübeck, Germany

*corresponding author; email address: marek.bartzik@uni-luebeck.de

***************************************************************************

The study was presented at the 2021 International Positive Psychology Conference (IPPA) and at the German conference of Work, Organizational and Business Psychology (AOW 2021), and will be part of the cumulative doctoral dissertation of Marek Bartzik.

***************************************************************************

ORCID of the authors:

**Marek Bartzik:** 0000-0002-2152-962X

**Fabienne Aust:** 0000-0002-6047-2961

**Corinna Peifer:** 0000-0002-6373-0866

**Ethics approval and consent for participants:** All procedures in this study were performed in accordance with the ethical standards of the German Psychological Society, of the 1964 Helsinki Declaration and its later amendments and were approved by the ethics committee of the Ruhr University Bochum (# 540, 612). Informed consent was obtained from all individual participants included in the study.

**Consent for publication:** Not applicable

**Availability of data and material (data transparency):** The datasets used and/or analysed during the current study are available from the corresponding author on reasonable request.

E-Mail: [marek.bartzik@uni-luebeck.de](mailto:marek.bartzik@uni-luebeck.de)

**Conflicts of interest/Competing interests** (include appropriate disclosures): all authors declare that there are no conflicts of interest.

**Funding information:**  The authors did not receive support from any organization for the submitted work.

**Author contribution statements:** All authors conceived the idea presented. M.B. carried out the experiment. M.B. and C.P. developed the theory. M.B. and F.A. wrote the methods section. F.A. and M.B. conducted the analyses and wrote the results section. All authors collaborated on the discussion section. All authors discussed the results and contributed to the final manuscript. C.P. supervised the conceptualization and process of writing the paper.

**Acknowledgements:** Not necessary

**Table S1**

*Self-constructed scales and items.*

| **German items used in the study** | **English items** | | **Scale** |
| --- | --- | --- | --- |
| **Stress – single item (self-constructed)** | | | |
| Wie sehr fühlten Sie sich gestresst? | How stressed did you feel? | | 1 = *not at all* to 5 = *very strong*  1 = *überhaupt nicht* bis 5 = *sehr stark* |
| **Flow Frequency Scale (Bartzik and Peifer, in preparation)** | | | |
| **Before the COVID-19 Pandemic**  Please imagine yourself in your daily work experience before the Corona Crisis, in early February 2020. Below are a series of questions about your daily experience at work.  Please indicate how often or rarely you had each experience. | | **During the COVID-19 Pandemic**  Please put yourself in your daily work experience during the Corona restrictions and answer the following questions:  Please indicate how often or rarely you had each experience. | |
| How often did you experience at work that... | | How often did you experience in your daily work during the Corona restrictions that... | |
| 1. … Sie vollständig auf Ihr Tun konzentriert waren. | 1. … you were completely focused on what you were doing. | | 1 = *Never*, 2 = *Almost never*, 3 = *Sometimes*, 4 = *Often*, 5 = *Very* *Often*, 6 = *(Almost) always*  1 = *Nie,* 2 = Fast nie, 3 = Manchmal, 4 = Häufig, 5 = Sehr häufig, 6 = *(Fast) immer* |
| 1. … Sie überrascht waren, wie schnell die Zeit verging. | 1. … you were surprised how quickly time passed. | |  |
| 1. … Sie im Tun Freude empfanden. | 1. … you felt joy in what you were doing. | |  |
| 1. … Sie ganz in einer Tätigkeit aufgingen. | 1. … you were completely absorbed in an activity. | |  |
| 1. … Sie genau im richtigen Maß gefordert wurden. | 1. … you were challenged in just the right degree. | |  |
| 1. … Sie die Arbeit genießen konnten. | 1. … you could enjoy the work. | |  |
| 1. … Sie Ihre Fähigkeiten optimal einsetzen konnten. | 1. … you could use your skills to the optimal extent. | |  |
| 1. … die Anforderungen wie für Sie gemacht waren. | 1. … the requirements were as made for you. | |  |
| 1. … sich die Arbeit gut anfühlte. | 1. … the work felt good. | |  |
| 1. … Sie im Tun glücklich waren. | 1. … you were happy in your doing. | |  |
| **Subscales - Flow Frequency Scale:**  *Absorption*: Item 1, Item 2, Item 4  *Perceived demand-skill balance*: Item 5, Item 7, Item 8  *Enjoyment*: Item 3, Item 6, Item 9, Item 10 | | | |
| **Satisfaction (self-constructed)** | | | |
| Alles in allem, wie zufrieden waren Sie mit Ihrer Arbeit? | All in all, how satisfied were you with your work? | | 1 = *extremely dissatisfied* to 7 = *extremely satisfied.*  1 = *sehr unzufrieden* bis 7 = *sehr zufrieden* |
| Alles in allem, wie zufrieden waren Sie mit Ihrem Leben? | All in all, how satisfied were you with your life? | |  |
| Alles in allem, wie zufrieden waren Sie mit Ihrer beruflichen Leistung? | All in all, how satisfied were you with your work performance? | |  |
| Alles in allem, wie zufrieden waren Sie mit Ihrem Wohlbefinden? | All in all, how satisfied were you with your well-being? | |  |
| **Appreciation (self-constructed)** | | | |
| Wie sehr fühlten Sie sich von Ihren Patienten wertgeschätzt? | How much did you feel appreciated by the patients? | | 1 = *not at all* to 5 = *very much.*  1 = *überhaupt nicht* bis 5 = *sehr stark* |
| Wie sehr fühlten Sie sich von der Gesellschaft wertgeschätzt? | How much did you feel appreciated by the society? | | 1 = *not at all* to 5 = *very much.*  1 = *überhaupt nicht* bis 5 = *sehr stark* |
| **COVID-19 pandemic items (self-constructed)** | | | |
| Ich machte mir aufgrund von Corona große Sorgen um meine eigene Gesundheit. | I was very concerned about my own health because of Corona. | | 1 = *do not agree at all* to 6 = *totally agree*  1 = *trifft überhaupt nicht zu* bis 6 = *trifft vollständig zu* |
| Ich machte mir aufgrund von Corona große Sorgen um die Gesundheit von Angehörigen und / oder Freunden. | I was very concerned about the health of family and friends because of Corona. | |  |
| Ich machte mir wegen Corona Sorgen um meine wirtschaftliche Zukunft. | I was very concerned about my economic future because of Corona. | |  |
| **Workload during COVID-19 pandemic (self-constructed)** | | | |
| Durch Corona hatte ich auf der Arbeit gerade… | Because of the COVID-19 pandemic I had to work… | | 1= *significantly less*, 2 = *less*, 3 = just the same, 4 = *more* or 5 = *significantly more*.  1= *deutlich weniger zu tun*, 2 = *weniger zu tun*, 3 = *gleich viel zu tun*, 4 = *mehr zu tun* oder 5 = *deutlich mehr zu tun*. |

**Table S2**

*Pearson correlations between sense of humor and psychological states before and during the COVID-19 pandemic*

| **Scales** | Sense of humor | Enjoyment of humor | Laughter | Verbal  humor | Finding humor in everyday life | Laughing at yourself | Humor under stress |
| --- | --- | --- | --- | --- | --- | --- | --- |
|  | **Before the COVID-19 pandemic** | | | | | | |
| Stress (single item) | .04 | .04 | .06 | .01 | .04 | -.00 | .02 |
| - Emotional irritation | **-.22**** | .00 | **-.23**** | **-.16*** | **-.21**** | **-.28**** | **-.23**** |
| - Emotional exhaustion | -.05 | .06 | **-.18*** | .03 | -.07 | **-.18*** | -.10 |
| Flow | **.25**** | .01 | **.32**** | .15 | **.27**** | **.27**** | **.26**** |
| - Absorption | **.20**** | .05 | **.27**** | .08 | **.23**** | **.16*** | **.21**** |
| - Demand-skill balance | **.22**** | -.00 | **.29**** | .10 | **.24**** | **.26**** | **.21**** |
| - Enjoyment | **.24**** | -.01 | **.30**** | **.18*** | **.25**** | **.26**** | **.23**** |
| Satisfaction |  |  |  |  |  |  |  |
| - Work | **.18*** | .01 | **.16*** | .13 | **.27**** | **.15*** | **.15*** |
| - Life | **.22**** | .02 | **.18*** | **.15*** | **.20*** | **.23**** | **.21**** |
| - Work performance | **.23**** | .05 | **.22**** | **.28**** | **.22**** | **.34**** | **.17*** |
| - Well-being | **.26**** | .01 | **.22**** | **.23**** | **.23**** | **.27**** | **.22**** |
| Appreciation |  |  |  |  |  |  |  |
| - Patients | .08 | -.04 | .15 | .07 | .15 | .10 | **.17*** |
| - Society | **.17*** | .13 | .15 | .10 | .07 | .06 | .13 |
|  | **During the COVID-19 pandemic** | | | | | | |
| Stress (single item) | -.08 | -.03 | -.03 | -.08 | .01 | -.14 | -.13 |
| - Emotional irritation | **-.17*** | -.13 | -.07 | -.11 | -.05 | **-.17*** | **-.25**** |
| - Emotional exhaustion | **-.19*** | -.12 | **-.20*** | -.10 | **-.16*** | **-.15*** | **-.27**** |
| Flow | **.35**** | **.22**** | **.33**** | **.22**** | **.34**** | **.22**** | **.43**** |
| - Absorption | **.29**** | **.22**** | **.27**** | **.23**** | **.31**** | **.16*** | **.40**** |
| - Demand-skill balance | **.30**** | **.18*** | **.31**** | .14 | **.26**** | **.20**** | **.33**** |
| - Enjoyment | **.36**** | **.21**** | **.29**** | **.25**** | **.34**** | **.23**** | **.43**** |
| Satisfaction |  |  |  |  |  |  |  |
| - Work | **.26**** | **.24**** | **.16*** | **.21**** | **.22**** | .09 | **.34**** |
| - Life | .15 | .06 | -.02 | **.23**** | .10 | .09 | **.22**** |
| - Work performance | **.17*** | .14 | .14 | **.21**** | **.18*** | **.16*** | **.31**** |
| - Well-being | **.18*** | .11 | .03 | **.20**** | **.19*** | .11 | **.27**** |
| Appreciation |  |  |  |  |  |  |  |
| - Patients | **.19*** | .14 | **.24**** | **.19*** | **.22**** | .11 | **.26**** |
| - Society | .14 | .12 | **.16*** | .13 | .11 | .07 | **.19*** |

*Note.* Significant results (two-tailed) are shown in bold face; ** *p* < .01 (two-tailed); * *p* < .05 (two-tailed). *n* varies due to the pairwise
deletion of data between 161 and 173.

**Table S3**

*Intercorrelations before the COVID-19 pandemic*

| **Scales** | 1 | 2 | 3 | 4 | 5 | 6 | 7 | 8 | 9 | 10 | 11 | 12 | 13 |
| --- | --- | --- | --- | --- | --- | --- | --- | --- | --- | --- | --- | --- | --- |
| Stress (single item) (1) | 1 |  |  |  |  |  |  |  |  |  |  |  |  |
| - Emotional irritation (2) | **.30**** | 1 |  |  |  |  |  |  |  |  |  |  |  |
| - Emotional exhaustion (3) | **.47**** | **.52**** | 1 |  |  |  |  |  |  |  |  |  |  |
| Flow (4) | **-.28**** | **-.46**** | **-.58**** | 1 |  |  |  |  |  |  |  |  |  |
| - Absorption (5) | **-.15*** | **-.36**** | **-.38**** | **.87**** | 1 |  |  |  |  |  |  |  |  |
| - Demand-skill balance (6) | **-.24**** | **-.39**** | **-.52**** | **.92**** | **.69**** | 1 |  |  |  |  |  |  |  |
| - Enjoyment (7) | **-.33**** | **-.48**** | **-.62**** | **.95**** | **.74**** | **.82**** | 1 |  |  |  |  |  |  |
| Satisfaction |  |  |  |  |  |  |  |  |  |  |  |  |  |
| - Work (8) | **-.41**** | **-.27**** | **-.52**** | **.57**** | **.40**** | **.52**** | **.60**** | 1 |  |  |  |  |  |
| - Life (9) | **-.19*** | **-.33**** | **-.33**** | **.36**** | **.27**** | **.34**** | **.35**** | **.37**** | 1 |  |  |  |  |
| - Work performance (10) | **-.19*** | **-.24**** | **-.38**** | **.36**** | **.23**** | **.35**** | **.38**** | **.50**** | **.32**** | 1 |  |  |  |
| - Well-being (11) | **-.23**** | **-.36**** | **-.35**** | **.47**** | **.38**** | **.43**** | **.46**** | **.39**** | **.55**** | **.28**** | 1 |  |  |
| Appreciation |  |  |  |  |  |  |  |  |  |  |  |  |  |
| - Patients (12) | **-.27**** | **-.28**** | **-.30**** | **.47**** | **.34**** | **.44**** | **.44**** | **.40**** | **.15*** | **.25**** | .14 | 1 |  |
| - Society (13) | **-.17*** | **-.20*** | **-.21**** | **.30**** | **.18*** | **.30**** | **.30**** | .14 | .15 | .06 | **.19*** | **.25**** | 1 |

*Note.* Significant results (two-tailed) are shown in bold face; ** *p* < .01 (two-tailed); * *p* < .05 (two-tailed). *n* varies due to the pairwise deletion of data between 161 and 174.

**Table S4**

*Intercorrelations during the COVID-19 pandemic*

| **Scales** | 1 | 2 | 3 | 4 | 5 | 6 | 7 | 8 | 9 | 10 | 11 | 12 | 13 |
| --- | --- | --- | --- | --- | --- | --- | --- | --- | --- | --- | --- | --- | --- |
| Stress (single item) (1) | 1 |  |  |  |  |  |  |  |  |  |  |  |  |
| - Emotional irritation (2) | **.55**** | 1 |  |  |  |  |  |  |  |  |  |  |  |
| - Emotional exhaustion (3) | **.58**** | **.59**** | 1 |  |  |  |  |  |  |  |  |  |  |
| Flow (4) | **-.33**** | **-.43**** | **-.62**** | 1 |  |  |  |  |  |  |  |  |  |
| - Absorption (5) | **-.20*** | **-.37**** | **-.44**** | **.87**** | 1 |  |  |  |  |  |  |  |  |
| - Demand-skill balance (6) | **-.23**** | **-.33**** | **-.50**** | **.89**** | **.68**** | 1 |  |  |  |  |  |  |  |
| - Enjoyment (7) | **-.39**** | **-.46**** | **-.67**** | **.93**** | **.72**** | **.74**** | 1 |  |  |  |  |  |  |
| Satisfaction |  |  |  |  |  |  |  |  |  |  |  |  |  |
| - Work (8) | **-.38**** | **-.45**** | **-.54**** | **.68**** | **.51**** | **.59**** | **.67**** | 1 |  |  |  |  |  |
| - Life (9) | **-.34**** | **-.35**** | **-.33**** | **.39**** | **.30**** | **.32**** | **.40**** | **.48**** | 1 |  |  |  |  |
| - Work performance (10) | **-.19*** | **-.37**** | **-.39**** | **.58**** | **.45**** | **.53**** | **.56**** | **.65**** | **.30**** | 1 |  |  |  |
| - Well-being (11) | **-.45**** | **-.39**** | **-.49**** | **.48**** | **.36**** | **.40**** | **.48**** | **.57**** | **.59**** | **.42**** | 1 |  |  |
| Appreciation |  |  |  |  |  |  |  |  |  |  |  |  |  |
| - Patients (12) | **-.19*** | **-21**** | **-.27**** | **.52**** | **.48**** | **.46**** | **.46**** | **.41**** | **.18*** | **.35**** | **.28**** | 1 |  |
| - Society (13) | -.06 | -.13 | **-.26**** | **.36**** | **.31**** | **.26**** | **.37**** | **.31**** | .08 | **.26**** | **.31**** | **.41**** | 1 |

*Note.* Significant results (two-tailed) are shown in bold face; ** *p* < .01 (two-tailed); * *p* < .05 (two-tailed). *n* varies due to the pairwise deletion of data between 166 and 174.

**Table S5**

*Buffering effects of sense of humor and appreciation using difference scores including sample sizes*

|  | *Stress* | | | *Emotional*  *Irritation* | | | | *Emotional Exhaustion* | | | | *Frequency of flow experience* | | | *Satisfaction –*  *Work* | | | *Satisfaction –*  *Life* | | | *Satisfaction –*  *Work performance* | | | *Satisfaction –*  *Well-being* | | | |  |
| --- | --- | --- | --- | --- | --- | --- | --- | --- | --- | --- | --- | --- | --- | --- | --- | --- | --- | --- | --- | --- | --- | --- | --- | --- | --- | --- | --- | --- |
|  | *n* | *R^2^* | β | | *n* | *R^2^* | β | | *n* | *R^2^* | β | *n* | *R^2^* | β | *n* | *R^2^* | β | *n* | *R^2^* | β | *n* | *R^2^* | β | | *n* | *R^2^* | β | |
| Appreciation |  |  |  | |  |  |  | |  |  |  |  |  |  |  |  |  |  |  |  |  |  |  | |  |  |  | |
| - Patients | 167 | .01 | -.11 | | 166 | .01 | -.09 | | 164 | **.06** | **-.25**** | 167 | **.09** | **.31**** | 166 | **.05** | **.22**** | 162 | .00 | -.05 | 161 | **.06** | **.23**** | | 161 | .00 | .02 | |
| - Society | 159 | .00 | -.04 | | 157 | .00 | -.03 | | 158 | .00 | -.03 | 161 | **.04** | **.19*** | 158 | .00 | .06 | 154 | .02 | -.14 | 153 | .00 | .01 | | 152 | .00 | .06 | |
| Sense of humor | 164 | .01 | -.11 | | 163 | .00 | -.02 | | 162 | **.04** | **-.20*** | 165 | **.03** | **.16*** | 163 | .02 | .13 | 159 | .00 | -.01 | 158 | .00 | .01 | | 156 | .00 | -.03 | |
| - Enjoyment of humor | 164 | .01 | -.08 | | 163 | **.04** | **-.20*** | | 162 | **.03** | **-.18*** | 165 | **.05** | **.23**** | 163 | **.05** | **.22**** | 159 | .01 | .07 | 158 | .01 | .12 | | 157 | .01 | .10 | |
| - Laughter | 163 | .01 | -.09 | | 162 | .01 | .10 | | 161 | .00 | -.06 | 164 | .00 | .03 | 162 | .00 | .04 | 158 | .01 | -.11 | 157 | .00 | -.01 | | 155 | .01 | -.11 | |
| - Verbal humor | 165 | .01 | -.08 | | 164 | .00 | .01 | | 163 | .02 | -.13 | 166 | .02 | .12 | 164 | .02 | .13 | 160 | .01 | .11 | 160 | .00 | .01 | | 157 | .00 | .01 | |
| - Finding humor in everyday life | 164 | .00 | -.03 | | 163 | .01 | .12 | | 163 | **.05** | **-.21**** | 166 | **.03** | **.16*** | 164 | .01 | .08 | 159 | .00 | -.03 | 159 | .00 | .05 | | 156 | .00 | -.02 | |
| - Laughing at yourself | 165 | .02 | -.14 | | 164 | .01 | .08 | | 163 | .00 | -.07 | 166 | .00 | .02 | 164 | .00 | .01 | 160 | .02 | -.12 | 159 | .01 | -.11 | | 158 | .01 | -.12 | |
| - Humor under stress | 168 | .02 | -.13 | | 167 | .00 | -.06 | | 166 | **.06** | **-.25**** | 169 | **.07** | **.26**** | 167 | **.06** | **.25**** | 163 | .01 | .11 | 163 | **.04** | **.20*** | | 160 | .01 | .07 | |

*Note.* Significant results are shown in bold face; ** *p* < .01; * *p* < .05.
